# Right ventriculography improves the accuracy of leadless pacemaker implantation in right ventricular mid-septum

**DOI:** 10.1007/s10840-022-01399-3

**Published:** 2022-10-25

**Authors:** Yaodong Li, Qiang Xing, Jiasuoer Xiaokereti, Cheng Chen, Jianghua Zhang, Xianhui Zhou, Yanmei Lu, Zukela Tuerhong, Baopeng Tang

**Affiliations:** 1 Cardiac Pacing and Electrophysiology Department, The First Affiliated Hospital of Xinjiang Medical University, No.137, South Liyushan Road, Xinshi Zone, Urumqi, Xinjiang China; 2 Xinjiang Key Laboratory of Cardiac Electrophysiology and Cardiac Remodeling, The First Affiliated Hospital of Xinjiang Medical University, Urumqi, China

**Keywords:** Micra™, Right ventriculography, Right ventricular septum, Cardiac computed tomography, Implantation

## Abstract

**Background:**

Implanting leadless pacemakers in the right ventricular (RV) apex is prone to causing pericardial tamponade and myocardial perforation.

**Objective:**

To investigate the feasibility and safety of right ventriculography-guided implantation of Micra™ leadless pacemaker (Micra™, Medtronic, Minneapolis, MN, USA) in the RV mid-septum.

**Methods:**

One hundred eight consecutive patients who underwent Micra™ implantation intended in the mid-septum were enrolled and randomized (3:1) into the radiography group (*n* = 81) with assistance of right ventriculography to illustrate the RV septum and the non-radiography group (*n* = 27). All subjects underwent a postoperative computed tomography (CT) scan to determine the Micra™ location. The Micra™ location assessed by CT image was compared between the two groups to confirm the accuracy of the intended pacing site. The duration of the procedure, X-ray radiation dose, and time were also compared between the two groups.

**Results:**

Reconstructed CT 3-D cardiac images found the Micra™ location in the intended mid-septum in 13 patients (48.1%, 13/27) in the non-radiography group and 76 patients (93.8%, 76/81) in the radiography group (*P* < 0.0001 between two groups). There was no significant difference in procedure interval between the two groups while the X-ray radiation dose (564.86 ± 112.44 vs. 825.85 ± 156.12 mGy, *P* < 0.0001), X-ray exposure time (7.79 ± 1.43 vs. 12.03 ± 2.86 min, *P* < 0.0001), and the number of fluoroscopy re-positioning (2.79 ± 1.03 vs. 6.41 ± 1.82, *P* < 0.0001) were significantly less in the radiography group than in the non-radiography group. No implantation-related complications were observed in both groups.

**Conclusion:**

Right ventriculography increases the accuracy of Micra™ implantation in the mid-septum and reduces X-ray exposure.

**Trial registration:**

The trial registration number (ChiCTR2100051374) and date (09/22/2021).

**Supplementary Information:**

The online version contains supplementary material available at 10.1007/s10840-022-01399-3.

## Introduction

Conventional transvenous pacemaker has been an effective therapy in patients with symptomatic bradycardia. However, implantation-related complications are not uncommon, including electrode dislocation, electrode fracture, venous thrombosis, tricuspid regurgitation, and, in particular, infection that usually requires a complete removal of the entire pacemaker system [1, 2]. The leadless pacemaker, on the other hand, overcomes these complications and its safety and efficacy have been reported in previous clinical studies [3–5]. However, there is uncertainty in the implantation site for the leadless pacemaker due to patients’ heart and anatomical variations and operators’ experience and preference. Previous studies have shown that mid-septal implantation of Micra™ leadless pacemaker (Micra™, Medtronic, Minneapolis, MN, USA) has advantages in terms of surgical safety and narrower paced QRS duration than implantation at the right ventricular (RV) apex where the procedure is more prone to causing cardiac perforation and RV apical pacing generates the widest QRS duration [6]. Previous clinical investigations of transvenous lead implantations have found that pacing in the mid-septum, when compared with the apical placement, significantly reduces the incidence of pericardial tamponade and myocardial perforation (52% vs. 33%, respectively) [7, 8]. However, there have been limited reports of clinical investigations on the mid-septal deployment of Micra™.

Recently, the technique of right ventriculography via contrast injection under fluoroscopy has been used to place the tip of a transvenous pacing lead in the RV septum [9]. The present study aimed to explore the technique of right ventriculography to locate a site in the RV mid-septum for deployment of a Micra™. The study objective was to assess the feasibility and safety of right ventriculography in guiding the mid-septal implantation of Micra™ in comparison with that without right ventriculography. Following Micra™ implantation, computed tomography (CT) scan was conducted to confirm the accuracy of the site of Micra™ in the RV mid-septal region. In addition, the study assessed electrical characteristics of pacing by Micra™ in septal locations.

## Methods

### Patients

A total of 108 consecutive patients who met class I or IIa indications for pacemaker therapy and intended to receive Micra™ implantation in the RV mid-septum were prospectively enrolled from December 18, 2019 to June 17, 2021. All patients were randomized to the radiography group (*N* = 81) or the non-radiography group (*N* = 27) in a 3:1 format. The 3:1 randomization was used for allowing more patients to receive mid-septal pacing. The exclusion criteria were as follows: (1) presence of thrombosis or cancer embolus in the inferior vena cava (IVC) or portal vein; (2) presence of bilateral femoral vein stenosis or tortuosity that could cause failure to accommodate the Micra™; (3) inability to tolerate heparinization or allergy to heparin; (4) presence of implanted devices that interfered with the Micra™, such as IVC filters or tricuspid mechanical valve replacement; (5) presence of severe renal insufficiency (glomerular filtration rate < 30 mL/min) or iodine allergy; (6) acute phase of myocardial infarction; and (7) contraindications for CT scan or contrast agents. All patients signed an informed consent form prior to the surgical procedure. This study was approved by the Ethics Committee of the First Affiliated Hospital of Xinjiang Medical University (ethical approval number: K201912-10) and registered in China Clinical Trial Registry (ChiCTR2100051374). The data that support the findings of this study are available from the corresponding author on reasonable request.

### Surgical implantation of Micra™

**Fig. 1:**
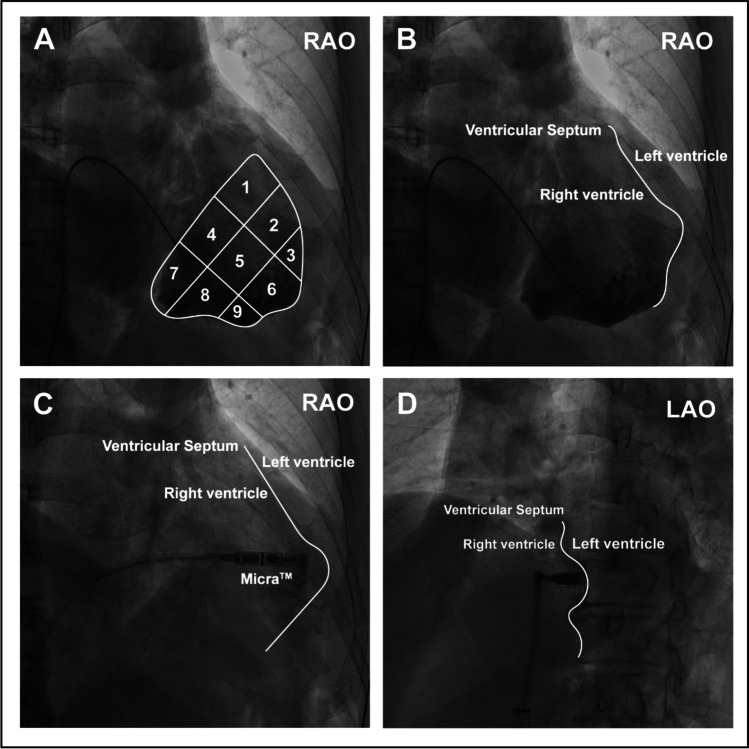
Right ventriculography under fluoroscopic RAO30 (**A**, **B**, and **C**) and LAO45 (**D**) in a patient. Nine septal subdivisions are depicted in the RAO30 image (**A**) following contrast injection that shows the RV septum (**B**). LAO45 (**D**) shows the separation (the green line) between RV and LV. The delivery catheter with Micra™ is shown in **C** and **D**

The procedure for Micra™ deployment has been well described before [10]. Briefly, patients were placed in a supine position and local anesthesia was administered at the right inguinal area. The femoral vein was punctured using the modified Seldinger technique and the Micra™ delivery catheter (Medtronic, Minneapolis, MN, USA) was inserted into the inferior right atrium via the IVC. The delivery catheter through the sheath was bent to cross over the tricuspid valve annulus to the right ventricle (RV). In the radiography group, the RV septum was visualized via the precise right ventriculography by the operator and the Micra™ was subsequently implanted in the mid-septum. The specific implantation steps are described below: a pigtail catheter (6Fr; TERUMO, Kyoto, Japan) was delivered along a sheath to the apex of the right ventricle, and the right ventriculography was achieved by injection of 20 cc contrast under fluoroscopic views in the right anterior oblique (RAO) 30° ± 10° and left anterior oblique (LAO) 45° ± 10° positions, respectively (Supplemental Materials Figure S1). The right ventriculography under fluoroscopic RAO30 showed the RV septum that was divided into nine subdivisions (named zones 1–9, Fig. 1A) and zone 2 (the mid-septum) was chosen as the initial intended implantation site for deployment of the Micra™ (Fig. 1C). In the non-radiography group, regular cap injection of 10 mL contrast was conducted that usually showed a small region of the right ventricle (Supplemental Materials Figure S1). In both groups, after transvalvular access to the right ventricle, the Micra was slightly rotated clockwise to point to the intended area in the fluoroscopic image of RAO 30, and the Micra™ was subsequently adjusted clockwise at the LAO view (usually at 40°). This was performed to ensure that the angle between the Micra™ and the spinal vertebrates was 70 to 90° and the height of the Micra™ was adjusted according to the spinal vertebrate positioning, which aided the anchoring of the Micra™ to the median septum.

If the pacing parameters at the chosen site were unacceptable, the distal delivery catheter was moved to a nearby location. After release of Micra™ was completed, electrical testing of the Micra™, including evaluations of pacing capture threshold, pacing impedance, and R-wave amplitude, was performed. Pacing and sensing electrical parameters were tested at a pulse width of 0.24 ms; the required capture threshold was ≤ 1.5 V, the pacing impedance ranged from 400 to 1500 Ω, and R-wave amplitude was ≥ 5 mV. After satisfactory test results, the row traction test verified that at least two hook teeth were fixed in the myocardium, then the tether was cut after pull and hold test and the delivery system was removed, and the puncture site was sutured for hemostasis. The duration of the procedure was defined as the time between the insertion of the femoral vein puncture needle into the skin and the withdrawal of the puncture sheath following pacemaker implantation.

### Data collection

Baseline data including demographic characteristics and indications for pacemaker implantation were collected at enrollment. Pacing parameters including capture threshold, R-wave amplitude, and pacing impedance were recorded at and post-implantation. The echocardiography parameters measured at enrolment and post-implantation were as follows: left ventricular ejection fraction (LVEF), left ventricular end-diastolic internal diameter (LVEDD), right ventricular size, and tricuspid valve regurgitation.

The procedural data collected at the implantation included the number of Micra™ delivery catheter repositioning (e.g., changes in the fluoroscopic projection and the distal part of the delivery catheter in search for a target location), length of surgery, X-ray exposure time and radiation dose, pacing parameters, and 12-lead electrocardiography (ECG) from which QRS duration and left ventricular activation time (the interval from pacing artifact to the peak of ECG V5 or V6) were measured. Adverse events were documented, including arteriovenous fistula, hematoma, incision site bleeding, persistent lymphatic fistula, vascular pseudoaneurysm, and other complications such as pericardial effusion and perforation, as well as leadless pacemaker displacement and embolization.

**Fig. 2:**
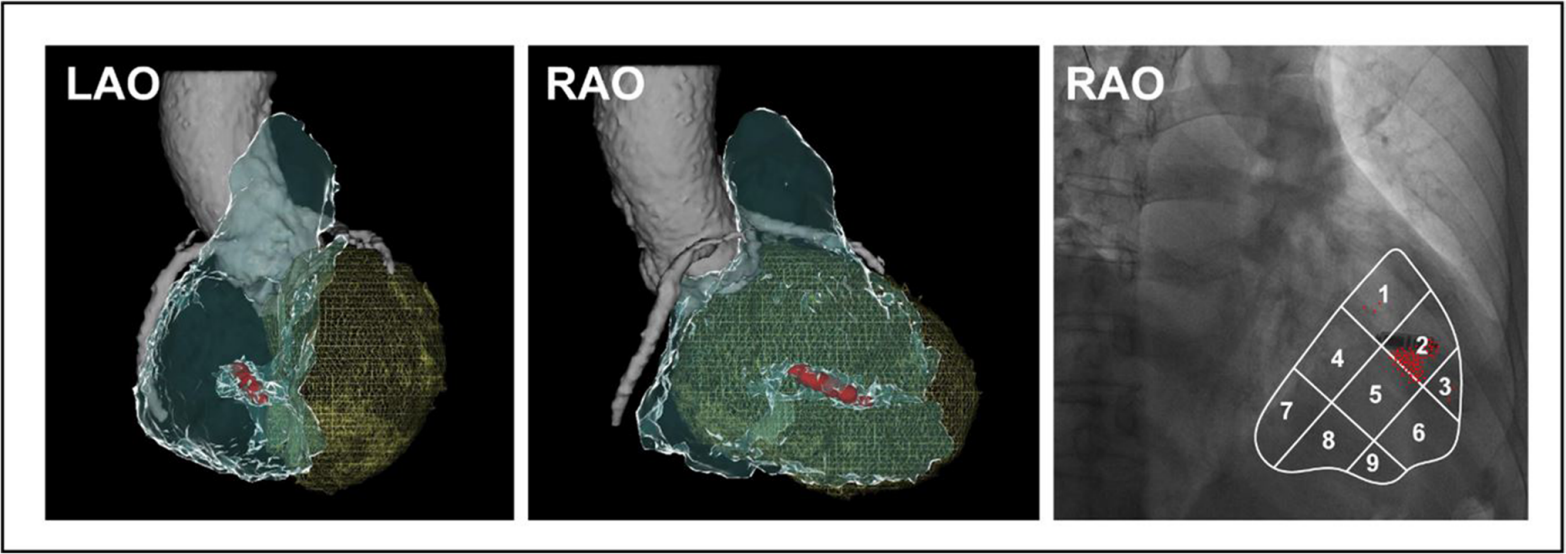
Micra™ implanted in the RV septum. Micra™ implanted in the mid-septum (zone 2 of the RV septum) in the CT-scan image in one patient (left: Micra™ in LAO 45; middle: Micra™ in RAO 30). Right panel: Scatter plot of Micra™ pacemakers distribution in the RV septum (RAO view) in the radiography group, with each red dot representing one patient

All patients underwent a computed tomography (CT) scan of the heart with contrast enhancement post-implantation (day 2 to day 30). Cardiac CT scan is a reliable method to identify the location of a pacing lead [11]. CT scan data were processed with 64 slides using the software Carto-Merge (Cartomerge™, Biosense Webster, Inc., Diamond Bar, CA, USA) to achieve 3-D cardiac reconstruction with Micra™ location, based on which the positional relationship between the Micra™ and the location in the right ventricular septal surface was determined (Fig. 2). The CT image-based pacing location was compared to the intended location by the operator at the implantation.

### Statistical analysis

All statistical analyses were performed using the SPSS 25.0 software. Categorical data were expressed as percentages and values, and numerical data with a normal distribution were expressed as mean ± standard deviation. Otherwise, median and interquartile range were used to express data with a skewed distribution. Quantitative data were compared using *t*-test, and analysis of variance (ANOVA) was performed for multiple comparisons among groups with post hoc tests. A two-sided test was used, and statistical significance was set at *P* < 0.05.

## Results

### Patient characteristics

A total of 108 consecutive patients were enrolled with 27 in the non-radiography group and 81 in the radiography group. All enrolled patients underwent successful implantation of a Micra™. Patient baseline characteristics and pacemaker indications were summarized in Table 1. Of 108 patients, 48 (44.4%) had sinus node dysfunction, 52 (48.1%) had II-III degrees of atrioventricular conduction block, and 8 (7.4%) had both sinus node dysfunction and atrioventricular conduction block. Overall, baseline LVEF and LVEDD were normal in both groups. The baseline ECG QRS duration was 91.0 ± 24.3 ms in the non-radiography group and 98.0 ± 24.3 ms in the radiography group (*P* = 0.206).

**Table 1:** Patient characteristics

Variable	Non-radiography (*n* = 27)	Radiography (*n* = 81)	*P*
Age (years)	66.04 ± 14.41	73.43 ± 10.56	0.005
Male sex (%)	19(70.4)	42(51.9)	0.093
BMI (kg/m^2^)	24.77 ± 4.68	24.59 ± 3.65	0.81
Hypertension (%)	15(55.6)	56(69.1)	0.198
Tricuspid regurgitation (%)	1(3.7)	6(7.4)	0.498
Diabetes mellitus (%)	9(33.3)	23(28.4)	0.626
Coronary artery disease (%)	11(40.7)	35(43.2)	0.822
CKD (%)	1(3.7)	2(2.5)	0.735
Hyperlipemia (%)	3(11.1)	1(1.2)	0.019
COPD (%)	6(22.2)	10(12.3)	0.211
Pulmonary arterial hypertension (%)	2(7.4)	23(28.4)	0.025
Previous pacemaker implantation (%)	1(3.7)	6(7.4)	0.498
Oral anticoagulants or antiplatelet			
Anticoagulation (%)	7(25.9)	11(13.6)	0.524
Antiplatelet (%)	9(33.3)	32(39.5)	
Anticoagulation + antiplatelet (%)	1(3.7)	4(4.9)	
Atrial fibrillation (%)	8(29.6)	24(29.6)	1
Pacemaker indication			
Sick sinus syndrome (%)	15(55.6)	33(40.7)	0.335
Second-degree AV block (%)	4(14.8)	14(17.3)	
Third-degree AV block (%)	1(3.7%)	16(19.8%)	
High AV block (%)	5(18.5%)	12(14.8%)	
Sick sinus syndrome and AV block (%)	2(7.4%)	6(7.4%)	
RV (mm)	18.33 ± 1.82	19.26 ± 2.75	0.106
LVEF (%)	62.61 ± 3.08	61.18 ± 3.41	0.057
LVEDd (mm)	47.89 ± 3.64	48.35 ± 4.49	0.625
LVESd (mm)	31.59 ± 2.58	32.87 ± 4.35	0.151

Abbreviations: *BMI*, body mass index; *CKD*, chronic kidney disease; *COPD*, chronic obstructive pulmonary disease; *AV*, atrioventricular; *RV*, right ventricular diameter; *LVEF*, left ventricular ejection fraction; *LVEDd*, left ventricular end-diastolic dimension; *LVESd*, left ventricular end-systolic dimension

### Implantation site

**Fig. 3:**
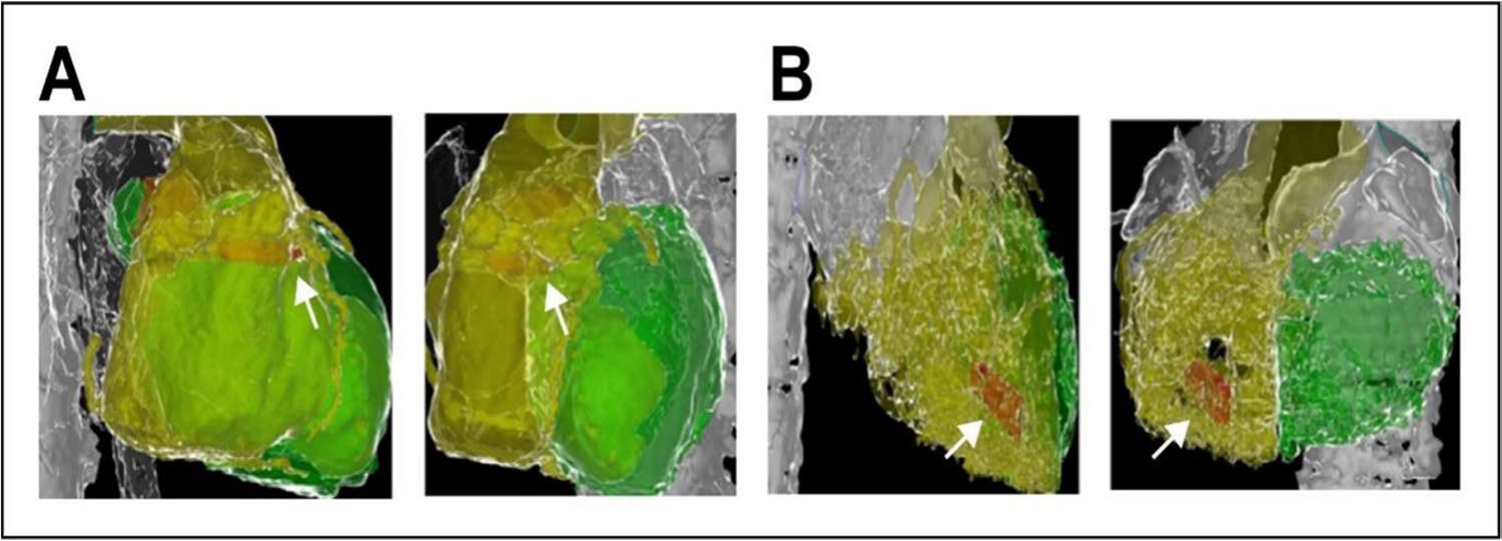
Examples of 3D CT reconstruction of the ventricles and interventricular septum, confirming that the Micra™ (white arrow) in the superior (**A**, left, RAO30; right, LAO45) and the inferior septum (**B**, left RAO30, right LAO 45). Yellow: right ventricle; green: left ventricle

During implantation, the implanter intended to deploy the Micra™ in the mid-septum based on the fluoroscopic RAO 30 image. Postoperative 3D reconstructive CT image of the heart was completed in all patients in both groups and the position of the Micra™ in the ventricular septum was determined. Figure 2 shows an example of Micra™ deployment in zone 2 of the mid-septum at implantation and confirmed in the post-implantation CT scan. Post-operative CT reconstructed images confirmed the Micra™ location in septal zone 2 (mid-septum) in 13 cases in the non-radiography group (48.1%, 13/27) and 76 cases in the radiography group (93.8%, 76/81, Fig. 2 and Table 2). The difference in the accuracy of intended deployment sites between the two groups was significant (*P* < 0.0001). Other locations confirmed by CT-scan images included the superior and inferior septum as shown in Fig. 3 and Table 2. In the non-radiography group, the Micra™ was deployed in the inferior septum in 6 patients (22.2%) and in the superior septum in 8 patients (29.6%) while in the radiography group, the Micra™ was deployed in the inferior septum in 2 patients (2.5%) and in the superior septum in 3 patients (3.7%). In comparison with fluoroscopic image, the site in the superior septum identified in the CT image was found in zone 1 of the fluoroscopic nine-partition image while the site in the inferior septum in the CT image was found in zone 3 that was close to the right ventricular apex.

**Table 2:** Distribution of Micra™ pacemaker locations in the RV septum

Location of Micra in the RV septum									
Zone	1	2	3	4	5	6	7	8	9
Radiography group (*N* = 81)	3	76	2	0	0	0	0	0	0
Non-radiography group (*N* = 27)	6	13	8	0	0	0	0	0	0

### Comparison of the procedure with or without right ventriculography

As shown in Table 3, the total procedural time was not significantly different between the two groups while the number of fluoroscopy repositioning for intended pacing sites was significantly higher in the non-radiograph group than in the radiograph group. Fluoroscopy repositioning was defined as a change in the target site under fluoroscopy before the deployment of the Micra™. Fluoroscopic use duration and the X-ray radiation dose were significantly greater in the non-radiography group than in the radiograph group.

**Table 3:** Operative duration and fluoroscopic use time

Variable	Non-radiography (*n* = 27)	Radiography (*n* = 81)	*P*
Total operative time (min)	43.85 ± 13.32	40.61 ± 15.82	0.34
Fluoroscopy duration (min)	12.03 ± 2.86	7.79 ± 1.43	< 0.0001
Number of fluoroscopy positions	6.41 ± 1.82	2.79 ± 1.03	< 0.0001
X-ray exposure (mGy)	825.85 ± 156.12	564.86 ± 112.44	< 0.0001

None of the study participants had procedure-related adverse events or complications such as abnormal pacing parameters, cardiac perforation and effusion, a prolonged hospital stay over 48 h, or infection. No worsening or development of tricuspid valve regurgitation and other implantation-related complications was observed by post-implantation echocardiograph and follow-up visits.

### Pacing parameters and 12-lead ECG characteristics

The intraoperative pacing capture threshold was moderately lower in the radiography group (0.48 ± 0.17 V @0.24 ms) than in the non-radiography group (0.58 ± 0.30 V @0.24 ms, *P* = 0.046). Location-based pacing capture threshold was 0.48 ± 0.16 V @0.24 ms in zone 2 of the septum, 0.62 ± 0.19 V @0.24 ms in the superior septum, and 0.25 ± 0.00 V @0.24 ms in the inferior septum (*P* = NS among three locations). Pacing impedance was 966.2 ± 260.9 in the non-radiograph group and 887.8 ± 234.6 Ω in the radiography group (*P* = 0.169 between two groups), and sensed R-wave amplitude was 10.6 ± 4.0 mV in the non-radiography group and 10.3 ± 4.3 mV in the radiograph group (*P* = 0.786 between two groups).

**Fig. 4:**
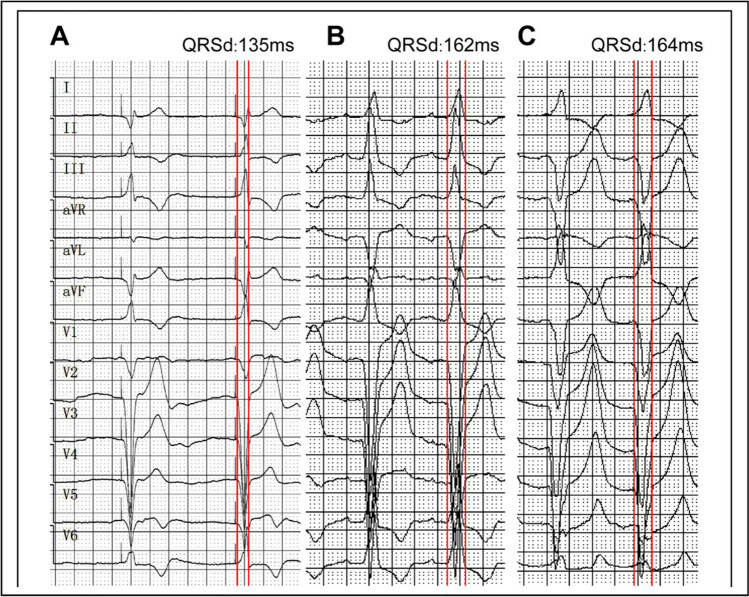
Example of location-based 12-lead ECGs. **A** Paced 12-lead ECG for Micra™ at zone 2 (the mid-septum). This is a transition of QRS waveform in precordial leads, e.g., from QS wave in V1 to R wave V6, consistent with the ECG characteristic of a typical septal pacing. Positive QRS in lead I/aVL associated with negative QRS complex in inferior leads, consistent with the ECG characteristic of an inferior septal pacing. **B** Paced 12-lead ECG for Micra™ at the superior septum with positive R wave in inferior leads and leads V5/V6 and QS type in leads V1–V4. **C** Paced 12-lead ECG for Micra™ pacemaker at the inferior septum with QRS complex of QS type in inferior leads and leads V1–V5, and rsR type in lead V6. QRSd, QRS duration measured between two red vertical bars

A relatively narrow paced QRS duration and short left ventricular activation time were observed in the radiography group and the non-radiography group (Table 4). Based on the location of the Micra™, the QRS duration and left ventricular activation time appeared slightly, but not statistically significantly, narrower and shorter for Micra™ locations in septal zone 2 (the mid-septum) than in other septal locations (Table 4). Figure 4 shows examples of the 12-lead ECG morphology in these three septal locations.

**Table 4:** ECG QRS duration (QRSd) and left ventricular activation time (LAVT)

Variable	Intrinsic QRSd (ms)	Paced QRSd (ms)	LVAT (ms)
Non-radiography group	93.55 ± 11.14	130.62 ± 11.59	99.62 ± 16.81
Radiography group	95.95 ± 15.57	122.79 ± 14.56	90.37 ± 15.65
Zone 2	96.63 ± 15.21	123.89 ± 13.75	91.79 ± 15.51
Superior septum	88.90 ± 7.54	126.18 ± 19.34	95.91 ± 18.41
Inferior septum	89.75 ± 11.69	132.37 ± 10.46	98.12 ± 22.99

There was no statistical difference between the radiography group and non-radiography group, and among three locations of zone 2, superior septum, and inferior septum

## Discussion

This prospective, randomized study assessed the feasibility of right ventriculography in assisting deployment of a Micra™ in the RV mid-septum and has several important findings. First, this technique yielded a high accuracy (93.8%) of the Micra™ deployment in the target location compared to the accuracy (48.1%) of the standard procedure. Second, with the assistance of right ventriculography the fluoroscopy exposure time and radiation dose were significantly reduced while the appropriate pacing parameters were preserved. No implantation-related adverse events were observed in both groups. Finally, septal pacing, especially in the mid-septum, provides a relatively narrow ECG QRS duration (123 ms in average).

The Micra™ Coverage with Evidence Development Study (the Micra CED) in 5746 patients implanted with Micra™ leadless pacemakers found that the incidence of complications following implantation was 3.3% in a 6-month follow-up period, in which myocardial perforation was the most common complication [11, 12]. Other complications included pericardial tamponade and anterior descending artery injury [7, 11, 13]. To reduce these implantation-related complications, the RV septum has become a target location for leadless pacemakers [6, 14]. The presence of more myocardial trabeculae and columnae carneae in the RV septum, particularly prominent in the middle of the septum, than in the RV apex and RV outflow tract makes the RV septum an ideal location for Micra™ leadless pacemakers with a less likely occurrence of myocardial perforation. In the Micra CED study, 52.1% of Micra™ leadless pacemaker locations were in the RV septum, 39.3% in the RV apex, 1.9% in the RV outflow tract, and 6.3% in junctions (including apex-septum and inferior septum) [11]. Sharma et al. [6] reported that 51.4% of leadless pacemaker implants were located in the ventricular septum, 28.6% in the right ventricular apex, and 20% in the RV outflow tract.

Currently, conventional fluoroscopic method is used in leadless pacemaker implantation. In the study by Hai et al. 15 in which multi-angle fluoroscopy was used to guide leadless pacemaker implantation in the mid-septum, 90% of the pacemaker implants were intended in the interventricular septum and 5% were in the right ventricular outflow tract under the RAO view. However, when left-sided fluoroscopy (LAO or left lateral view) was performed, the actual implantation site was observed at the anterior free wall in 17.6% of patients with the intended location in the septum. Therefore, developing an accurate and effective method for implanting leadless pacemakers in the mid-septum remains a challenge.

The present study observed a higher accuracy of the Micra™ deployment at the septal target location with assistance of right ventriculography than that without right ventriculography. Furthermore, the present study demonstrated not only the safety of the technique but also a significant reduction in X-ray exposure time and radiation dose. The less fluoroscopic use time in the radiography group was likely because the operator could directly manipulate the distal part of the delivery catheter against the mid-septum according to the right ventriculography while in the non-radiography group the operator used more time and fluoroscopy repositioning to figure out the target site. The implantation site in the inferior septum is close to the RV apex, which may have a high risk of cardiac perforation while the septal superior site has fewer myocardial trabecula, which can be difficult in the leadless pacemaker deployment. In consideration of the effects of right ventricular anatomic variants and cardiac transposition on the procedure of leadless pacemaker implantation, right ventriculography provides more detailed information on the right ventricular contours and anatomic landmarks in the fluoroscopic LAO and RAO positions. Right ventriculography may also be used to identify the septal tangent and position of the tricuspid valve annulus. Additionally, right ventriculography can show the specific anatomic IVC and heart structural variants and allow easier access to the right ventricle with guidewires and sheaths. Right ventriculography has previously been used to identify the target site in the RV mid-septum for the transvenous lead deployment for left bundle branch pacing and His bundle pacing [16]. Therefore, right ventriculography can be a useful technique to facilitate the deployment of a Micra™ or a transvenous pacing lead at an intended location.

Conventional RV apical pacing causes a significant wide ECG QRS duration and interventricular dyssynchrony that are associated with an increased risk of cardiac dysfunction in patients with frequent pacing. Pursuit of more physiological pacing method is continuing. Previously, septal pacing has been proposed as an alternative to RV apical pacing though there has been no conclusive clinical evidence. The true location of the pacing lead tip using fluoroscopy is often inaccurate, leading to conflicting results of septal pacing in acute and chronic studies. Recently, the study by Sharma et al. demonstrated that a mid-septal pacing by Micra™ resembles physiological pacing and tends to achieve a relatively narrow paced QRS interval [6]. Our study also found that pacing by the Micra™ at the mid-septal location generated a relatively narrow QRS duration and left ventricular activation time (Supplemental Material Figure S2, a comparison in 12-lead ECG between RV septal pacing vs. apical pacing). Currently, left bundle branch pacing has been considered as a physiological pacing modality [17–19]. To achieve left bundle branch pacing, the pacing lead tip is screwed into the left side of the septum from the right side of the RV mid-septum [20]. In the future, the leadless pacemaker with a modified long pacing electrode and assisted with RV ventriculography for the target entry site in the RV septum can also achieve left bundle branch pacing.

### Study limitations

The study was performed in a single center with relatively small patient numbers and in a non-blinded procedure that might introduce potential bias. The study could not address the potential difference in safety between the ventriculography group and the non-ventriculography group because the operator made the effort to place the leadless pacemaker in the RV septum in both groups. While right ventriculography could clearly show a full picture of the RV septum, the sites of Micra location appeared clustered in zone 2 of the RV septum, not other mid-septal regions such as zone 5. This was because the distal part of the Micra delivery catheter that was inserted via the IVC could not be appropriately bent against zone 5 of the mid-septum. Thus, re-designing delivery catheter may be necessary for a pacing site chosen from a wide range. The generalization of the technique of right ventriculography in routine clinical practice needs further validation in a multi-center study with a large population and diverse pacing indications. Furthermore, long-term clinical outcomes with this approach and pacing in the mid-septum remain unknown though the present study found a relatively narrow QRS duration and a recent report showed a possibility of leadless pacemaker in prevention of pacing-induced cardiomyopathy [21]. Thus, clinical studies are needed to evaluate the long-term clinical effects of Micra™ implanted in the mid-septum in comparison with the pacing site in the RV apical septum or apex.

## Conclusion

In the present study, use of right ventriculography yielded a high success and accuracy rate to deploy a Micra™ in the mid-septum with reduced time in X-ray exposure and without compromising safety. Septal deployment of the Micra™ has a potential to reduce complications such as perforation, effusion, and pericardial tamponade when compared to the implantation site at the RV apex. Thus, right ventriculography to guide Micra™ implantation in the mid-septum can be a useful method, especially for beginners, less experienced centers, and patients with specific cardiac anatomical variations in a routine clinical practice.

## Supplementary Information

Below is the link to the electronic supplementary material.

Supplementary file1 (DOCX 589 KB)
