# Supplementary material for: Right ventriculography improves the accuracy of leadless pacemaker implantation in right ventricular mid-septum
Source: J Interv Card Electrophysiol. 2022 Oct 25;66(4):941–9. doi: 10.1007/s10840-022-01399-3 (PMC10173092; doi:10.1007/s10840-022-01399-3)

**Supplemental Figure S1.** Visualization of the right ventricular (RV) septum by contrast injection via a pigtail catheter or the cap of a leadless pacemaker delivery catheter in two patients. The RV septum in both RAO and LAO fluoroscopic images is clearly better for the pigtail catheter injection than by the cap injection. The cap injection of contrast only shows a local region of the RV while the pigtail catheter injection reveals the whole right ventricle (e.g., right ventriculography). Thus, the study used the pigtail catheter injection in the radiography group.


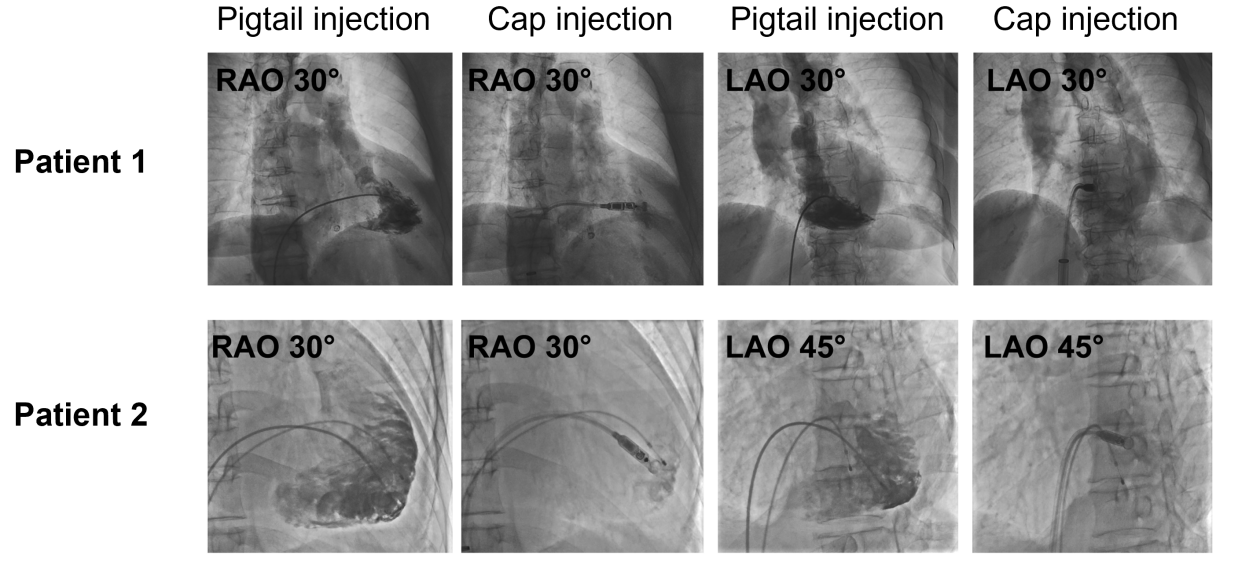


**Supplemental Figure S2**. Example of 12-lead ECG during pacing by the Micra™ at mid-septum (A) in a patient and during right ventricular apical pacing in another patient (B). ECG QRS duration appears shorter in septal pacing (132 ms) than in RV apical pacing (149 ms). Lack of a notch in R wave in ECG leads I and V5/6 suggests on obvious left bundle branch block as frequently observed in right ventricular apical or free-wall pacing. A horizontal bar represents 200 ms, the space between two red vertical line indicates QRS duration.


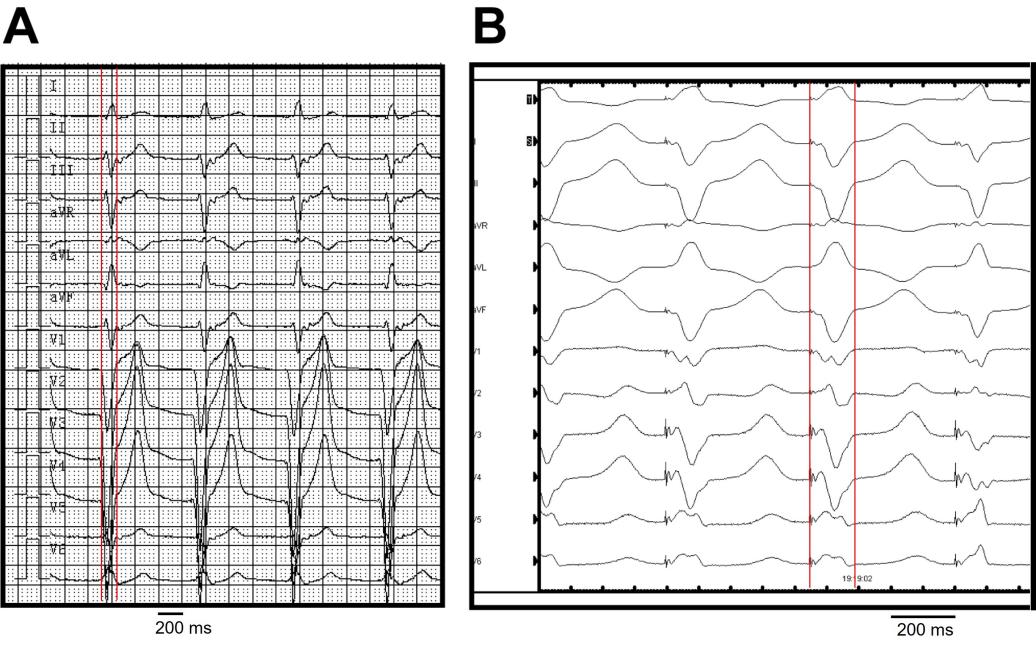

Supplement: Supplementary file 1 — Supplementary file1 (DOCX 589 KB) [file 10840_2022_1399_MOESM1_ESM.docx]
